# A critical reappraisal of vasopressin and steroids in in-hospital cardiac arrest

**DOI:** 10.1186/s13054-024-04962-8

**Published:** 2024-06-06

**Authors:** Spyros D. Mentzelopoulos, Athanasios Chalkias

**Affiliations:** 1 First Department of Intensive Care Medicine, Medical School, National and Kapodistrian University of Athens, Athens, Greece; 2 Department of Intensive Care Medicine, Evaggelismos General Hospital, 45-47 Ipsilandou St, 10675 Athens, Greece; 3 Institute for Translational Medicine and Therapeutics, University of Pennsylvania Perelman School of Medicine, Philadelphia, PA 19104-5158 USA; 4 Outcomes Research Consortium, Cleveland, OH 44195 USA

## Epinephrine during resuscitation

Advanced life support (ALS) objectives include maximization of coronary perfusion pressure (CPP) for prompt return of spontaneous circulation (ROSC) and minimization of cardiac arrest-associated ischemia–reperfusion injury. Epinephrine, the standard ALS vasopressor, improves ROSC rate, with uncertain effect on neurological outcome [1]. Epinephrine efficacy is limited by its potential to cause arrhythmias, myocardial ischemic contracture, and cerebral microcirculatory dysfunction [2].

## Combined stress-hormone approaches

Effectiveness of stress-hormone interventions may depend on timely administration and/or dose for prompt onset of action, and/or use of concurrently administered combinations. In a recent, two-center, randomized clinical trial (RCT) of in-hospital cardiac arrest (IHCA; participants, n = 184) [3], we assessed the effect of methylprednisolone 40 mg, or placebo (plus repeated 1-mg epinephrine) during ALS, followed by postresuscitation hydrocortisone (240 mg/day for 7 days maximum and gradual taper) or placebo on several physiological and long-term outcomes. Neutral results, primarily on early post-ROSC arterial pressure and inflammatory response, strongly implied a resistance to previously well-documented, circulatory and immunomodulatory effects of steroids [3]. Nevertheless, single, high-dose (i.e., 250 mg) methylprednisolone within 5–30 min following successful out-of-hospital ALS has been recently associated with lower postresuscitation interleukin-6, improved postresuscitation hemodynamics and higher probability of survival to discharge [4, 5]; these benefits might reflect a rapid, nongenomic, high-dose methylprednisolone action [4].

A major characteristic of our IHCA-steroids RCT was prolonged median ALS duration, i.e. 25–27 min [3]. Notably, pooled data analyses (n = 368) from our two, prior vasopressin-steroids-epinephrine (VSE) RCTs [6, 7] revealed median ALS duration of just 14 min in VSE patients, as opposed to 20 min in controls [3]. In these RCTs, we added up to five doses of 20-IU vasopressin to epinephrine during ALS to maximize CPP/expedite ROSC by concurrent stimulation of V1A vasopressin and alpha-1 adrenergic receptors [2, 6, 7]; stress-dose steroids were also given during and after ALS for their hypertensive/anti-inflammatory effects. Triple stress-hormone intervention resulted in higher ROSC-rates and postresuscitation arterial pressure, lower serum cytokine concentrations, more organ failure-free days, and improved long-term outcomes [6, 7].

A follow-up, Danish RCT (n = 501) testing vasopressin, adrenaline and methylprednisolone (VAM) during ALS (without postresuscitation steroids) reported improved ROSC-rate and neutral results on long-term outcomes [8]. The results of an individual patient-data meta-analysis (IPDMA) including all 3 RCTs were inconclusive [9], supporting a suggestion against VSE/VAM in IHCA [10]. However, following correction of misclassification of a VSE-2 study participant [7], IPDMA’s adjusted odds ratio (aOR) (95% confidence interval (CI) for cerebral performance category (CPC) score ≤ 2 at discharge rose to 1.80 (1.08–3.01), in favor of VSE/VAM [9].

Correction-associated, main changes in IPDMA results are summarized in Table 1. Key pertinent messages include (1) frailty/fragility of results’ positivity, depending on minor changes in a small absolute difference of approximately 4% in favorable neurological outcome; and (2) lack of power of included RCTs to detect differences in favorable neurological outcome at a level of 4–5%.

**Table 1:** Correction-induced changes in an IPDMA of three RCTs of vasopressin and steroids in cardiac arrest

	Corrected descriptive data intervention vs. control	Original IPDMA aOR (95%CI)	Corrected IPDMA aOR (95%CI)
Survival to discharge with CPC of 1 or 2	42/415 (10%) vs. 28/454 (6%)	1.64 (0.99–2.72)	1.80 (1.08–3.01)

	Bayesian prior beliefs	Original Bayesian posterior mean OR (95% CRI)	Corrected Bayesian posterior mean OR (95% CRI)
Survival to discharge with CPC of 1 or 2	Non-informative	1.65 (0.91–2.45)	1.82 (1.09–3.09)
	Moderate optimistic	1.33 (0.96–1.71)	1.37 (1.03–1.85)
	Weak optimistic	1.52 (0.90–2.18)	1.57 (1.05–2.36)
	Weak skeptical	1.54 (0.90–2.31)	1.63 (1.03–2.63)

IPDMA, individual patient data meta-analysis; RCT, randomized clinical trial; CPC, cerebral performance category; aOR, adjusted odds ratio; CI, confidence interval; CRI, credible interval

Major, corrected IPDMA results were primarily driven by our RCTs [9], which had key differences from the Danish trial [2], as further detailed in Table 2. Regarding time-to-study drugs (T_DRUG_), IPDMA data correction revealed significant effect measure modification, with decremental T_DRUG_ of ≤ 6 min, favoring VSE/VAM as regards survival to discharge and CPC score ≤ 2 at discharge [9]. Danish trial subgroup point estimates for both ROSC and 30-day survival/neurological outcome were also favorable for VAM (ranging within 1.17–1.46) at T_DRUG_ ≤ 8 min [8], implying a T_DRUG_-dependent, favorable response to VAM in 251/501 (50%) of study participants.

**Table 2:** Key differences between the Greek VSE trials and the Danish VAM IHCA trial

Key characteristic	VSE 1 and 2—pooled data	VAM-IHCA (Danish trial)	Potential effect
Time to study drugs, median (IQR)—min	4 (3–5)	9 (6–12)	Earlier, simultaneous activation of V1A-vasopressin and α1-adrenergic receptors, with maximization of pressor effect and associated probability of prompt ROSC,^a^ likely occurring only in intervention groups (vs. control) of the VSE 1 and 2 RCTs
Epinephrine and Vasopressin started and always given simultaneously	Yes	No	
Median time lag between first dose of epinephrine and study drugs (min)	0	3–4	
Significantly shorter median (IQR) time to ROSC (min) in intervention group(s) versus control	Yes; 14 (7–24) versus 20 (10–30)—*P* < 0.001	No; 16 (12–25) versus 18 (11–31)—*P*-value NR	Attenuated ischemia / reperfusion injury and lower risk of epinephrine-related adverse effects likely present only in intervention groups (vs. control) of the VSE 1 and 2 RCTs
Significantly lower median (IQR) total dose (mg) of epinephrine in intervention group(s) versus control	Yes; 4 (2–6) versus 5 (3–9)—*P* < 0.001	No; 3 (2–5) versus 3 (2–5)—*P*-value NR	
SAP < 90 mmHg within 20 min of ROSC, intervention group(s) versus control (%)	19% versus 44%—*P* < 0.001^b^	NR but significant difference unlikely ^c^	Early postresuscitation hypotension has been consistently associated with increased in-hospital mortality [11]; reported differences may partly explain the long-term benefit observed only in the VSE 1 and 2 RCTs
Lowest MAP ≤ 50 mmHg and SAP ≤ 80 mmHg, intervention group(s) versus control (%)	17% versus 28%—*P* = 0.12 and 15% versus 29%; *P* = 0.03^d^	NR but significant difference unlikely^c^	
Postresuscitation use of steroids in intervention group(s) versus control (%)	99% versus 22% ^d^	26% versus 46% ^e^	In VAM-IHCA, there was non-protocolized, more frequent use of potentially beneficial interventions in the control group
Postresuscitation use of ECMO in intervention group(s) versus control (%)	0% versus 0% ^d,f^	14% versus 30% ^e^	

VSE, vasopressin-steroids-epinephrine; VAM, vasopressin-adrenaline-methylprednisolone; IHCA, in-hospital cardiac arrest; IQR, interquartile range; ROSC, return of spontaneous circulation; NR, not reported; RCT, randomized clinical trial; SAP, systolic arterial pressure; MAP, mean arterial pressure; ECMO, extracorporeal membrane oxygenation

Reported data originate from the total of the VSE 1 and 2 or VAM-IHCA participants, unless otherwise specified. For data reported as median (IQR), *P*-values were determined using the Mann Whitney U test. For data reported as percentages, *P*-values were determined by Fisher’s exact test

^a^, Defined as ROSC after ≤ 3 vasopressor doses

^b^, VSE 1 and 2 data originate from the pooled subgroup of survivors for ≥ 4 h with postresuscitation shock and available SAP data (intervention, n = 94; control, n = 72)

^c^, Analyses of VAM-IHCA hemodynamic data revealed very similar, early postresuscitation arterial pressures and vasopressor support [2]; consequently, there was likely no significant, between-group difference in the frequency of postresuscitation hypotension

^d^, VSE 1 and 2 data originate from the pooled subgroup of survivors for ≥ 24 h (intervention, n = 94; control, n = 69)

^e^, VAM-IHCA data originate from survivors for ≥ 24 h (intervention, n = 63; control, n = 61)

^f^, There was no postresuscitation use of ECMO in the VSE 1 and 2 studies

## Multi-level VSE effects in IHCA

Transcriptional signaling by glucocorticoids is limited by proteasome degradation of the phosphorylated (oxidized) glucocorticoid receptor (GR) [3, 12]. During ischemia and reperfusion, proteasome mediates removal of oxidized, intracellular proteins [12]. Furthermore, the longer the duration of ischemia, the greater the degradation of adenosine 5′-triphosphate and intracellular accumulation of hypoxanthine [13]. During subsequent reperfusion, hypoxanthine is reconverted to xanthine, by xanthine oxidase, with concurrent production of toxic, reactive oxygen species [12]. Consequently, prolonged ischemia time (followed by reperfusion) is associated with greater, intracellular, oxidative stress [13], and likely, more extensive oxidation and loss of function [12] of various proteins, including the GR. Accordingly, a recent study reported rapid, post-ROSC decline in B/T lymphocyte GR-expression [14].

In our trials [6, 7], VSE patients received post-ROSC stress-dose hydrocortisone (300 mg/day for 7 days maximum and gradual taper). Furthermore, the shorter "low-flow/ischemia" time might have mitigated the ischemia–reperfusion induced oxidation/proteasome degradation of GR, with consequent preservation of vasopressor and anti-inflammatory effects of steroids. These two pharmaco-physiological factors explain the decreased frequency of potentially detrimental, early, postresuscitation hypotension in VSE patients (Table 2). Also, in our trials, steroid treatment was associated with lower postresuscitation cytokine concentrations [6]. This indicates attenuation of cardiac arrest-associated systemic inflammatory response, and partly explains the lesser organ dysfunction in VSE groups [6, 7].

## Additional key facts supporting VSE use in IHCA

IHCA nonshockable rhythms’ incidence and long-term outcomes are comparably high and poor (respectively) across Registry studies and control groups of VSE/VAM RCTs [Table 3; 6–8; Additional file 1]. Relatively minor differences can be partly explained by potentially more frequent pseudo-pulseless electrical activity (PEA) (which has better prognosis) in studies with high, overall incidence of PEA; also, in Registry studies, epinephrine was not given to some patients and this was associated with improved survival (Table 3; Additional file 1). Thus, our combined VSE 1 and 2 group results of nonshockable rhythms’ survival to discharge and CPC ≤ 2 at discharge of 17% and 14% (respectively) [6, 7] suggest VSE benefit in the large subgroup of nonshockable IHCA. Accordingly, corrected IPDMA aOR (95%CI) of VSE/VAM versus control for CPC score ≤ 2 at discharge was 2.02 (1.11–3.67) [9].

**Table 3:** Comparative presentation of frequency/outcomes of nonshockable presenting rhythms across studies, and of do-not-resuscitate practices across countries of study conduct

	US Registry studies 1999–2010 (n = 44,567–84,625)	Greek VSE RCTs^a^ (2006–2010)		Danish VAM RCT (2018–2021)	
		Control	VSE	Control	VAM
		(n = 190)	(n = 178)	(n = 264)	(n = 237)
Nonshockable rhythms Total, n (%) PEA, % Asystole, %	**36,344–67,135 (76–82)** 37–49^b^ 30–40	**160 (84)** 17 67	**149 (84)** 20 64	**233 (88)** 52 36	**216 (92)** 57 35
Cardiac arrest location					
ICU / CCU / PACU – OR, %	48–59	40	40	10	12
Monitored unit / area / ward, %	20–25	0	0	36	25
Emergency department^c^, %	11	16	16	14	8
Other^d^, %	5	0	0	15	14
Monitored – total, n (%)	**35,925–68,064 (68–85)**	**75** (**40)**	**71 (40)**	**121 (46)**	**87 (37)**
Monitored, % of PEA^c^	87	38	50	NR	NR
Monitored, % of asystole^c^	79	39	40	NR	NR
Nonmonitored unit / area / ward – total, n (%)	**7748–16,561 (15–21)**	**115 (61)**	**107 (60)**	**145 (54)**	**150 (63)**
Nonmonitored, % of PEA^c^	13	63	50	NR	NR
Nonmonitored, % of asystole^c^	21	61	60	NR	NR
Witnessed cardiac arrest^c^, n (%)	**79**	**89**	**89**	**77**	**71**
Time to resuscitation team arrival (min)^e^	**NR**	**2 (1–3)**	**2 (1–3)**	**3 (2–4)**	**3 (2–4)**
Time to rhythm analysis, (min)^f^	**NR**	**3 (2–4)**	**3 (2–4)**	**2 (1–4)**	**2 (1–4)**
Nonshockable rhythms survival to hospital discharge, %^g,h^	**7–16**	**5**	**17**	**10**	**8**
Nonshockable rhythms CPC score ≤ 2 at discharge, %^g,h^	**6–12**	**3**	**15**	**6**	**5**

	US Registry study on nonshockable rhythms 2006–2019 (n = 227,097)	Greek VSE RCTs^a^ nonshockable rhythms		Danish Registry study on nonshockable rhythms 2017–2018 (n = 2780)
		Control (n = 160)	VSE (n = 149)	
General factors associated with asystole/PEA^i^				
Monitored—total, n (%)	**188,949 (83)**	**62 (39)**	**63 (42)**	**1093 (39)**
Monitored PEA, % of total	67	19	29	65
Monitored asystole, % of total	33	81	71	35
Nonmonitored—total, n (%)	**38,148 (17)**	**98 (61)**	**86 (58)**	**1687 (61)**
Nonmonitored PEA, % of total	54	20	21	47
Nonmonitored asystole, % of total	46	80	79	53
Time to rhythm analysis – total (min)^f^	**NR**	**3 (2–4)**	**3 (2–4)**	**NR**
Time to rhythm analysis in PEA (min)^f^	NR	2 (2–3)	2 (2–4)	2 (0–4)
Time to rhythm analysis in asystole (min)^f^	NR	3 (2–4)	3 (2–4)	3 (1–5)
Cardiac arrest within 23.00–07.00 h-total, n (%)	**71,321 (31)**	**51 (32)**	**50 (34)**	**934 (34)**
Cardiac arrest within 23.00–07.00 h, % of PEA	30	28	39	28
Cardiac arrest within 23.00–07.00 h, % of asystole	35	33	32	40
Witnessed cardiac arrest – total, n (%)	**195,583** **(86)**	**140 (88)**	**132 (89)**	**2078 (75)**
Witnessed cardiac arrest, % of PEA	89	91	89	86
Witnessed cardiac arrest, % of asystole	80	87	89	61
Patient factors associated with asystole/PEA^k^				
Age – total (years)	**65 (16)**	**72 (58–78)**	**68 (53–77)**	**NR**
Age – PEA (years)	65 (16)	73 (62–78)	73 (60–80)	74 (65–81)
Age – asystole (years)	66 (16)	70 (57–78)	68 (52–77)	75 (66–82)
Sex – total—female, n (%)	**96,826 (43)**	**61 (38)**	**47 (32)**	**1077 (39)**
Sex—PEA – female, %	42	34	19	37
Sex – asystole – female, %	43	39	35	41
Overweight/Obesity – total, n (%)^l^	**NR**	**70 (44)**	**65 (44)**	**NR**
Overweight/Obesity, % of PEA	NR	38	47	NR
Overweight/Obesity, % of asystole,	NR	45	43	NR
COPD – total, n (%)	**NR**	**40 (25)**	**30 (20)**	**500 (18)**
COPD, % of PEA	NR	25	25	17
COPD, % of asystole	NR	25	19	20
Gastrointenstinal cancer – total, n (%)	**NR**	**7 (4)**	**5 (6)**	**117 (4)**
Gastrointenstinal cancer, % of PEA	NR	3	6	3
Gastrointenstinal cancer, % of asystole	NR	5	4	5
Ischemic heart disease – total, n (%)	**NSR**	**55 (34)**	**52 (35)**	**599 (22)**
Ischemic heart disease, % of PEA	NSR	44	36	24
Ischemic heart disease, % of asystole	NSR	32	35	19
Intubation before cardiac arrest – total, n (%)	**100,875 (44)**	**98 (61)**	**73 (49)**	**245 (9)**
Intubation before cardiac arrest, % of PEA	47	67	53	11
Intubation before cardiac arrest, % of asystole	40	60	48	6
Duration of resuscitation or ALS (min)^m^				
Duration of resuscitation/ALS in PEA,	20 (20)	20 (8–34)	14 (7–23)	NR
Duration of failed resuscitation/ALS in PEA	30 (23)	35 (24–60)	26 (15–40)	NR
Duration of resuscitation/ALS in asystole	20 (19)	20 (10–30)	15 (7–27)	NR
Duration of failed resuscitation/ALS in asystole	26 (22)	30 (20–33)	34 (19–50)	NR
Failed resuscitation—total, n (%)	**91,133 (40)**	**66 (41)**	**26 (17)**	**1632 (59)**
Failed resuscitation PEA, % of total	62	17	39	47
Failed resuscitation asystole, % of total	38	83	61	53
Monitored—total, n (%)	**NR**	**20 (30)**	**7 (27)**	**NR**
Monitored PEA, % of total	NR	10	57	NR
Monitored asystole, % of total	NR	90	43	NR
Nonmonitored—total, n (%)	**NR**	**46 (70)**	**19 (73)**	**NR**
Nonmonitored – PEA, % of total	NR	20	32	NR
Nonmonitored – asystole, % of total	NR	80	68	NR
Data on country-level resuscitation practices	**US**	**Greece**		**Denmark**
Do-not-resuscitate decisions legally supported and routinely documented in patient records^n^	**Yes**	**No**		**Yes**
Reported rates of failed resuscitation among ICU decedents, %^n,o,p^	**7**	**42**		**1**

Fractional data and proportions are reported as number (percentage) or percentage, respectively; continuous data are reported as mean (SD) or median (IQR), depending on distribution normality. Major/summary data are highlighted in bold script.

US, United States; VSE, vasopressin-steroids-epinephrine; VAM, vasopressin-adrenaline-methylprednisolone; ICU, intensive care unit; CCU, coronary care unit; PACU-OR, postanesthesia care unit – operating room; RCT, randomized clinical trial; PEA, pulseless electrical activity; NR, not reported; CPC, cerebral performance category; COPD, chronic obstructive pulmonary disease; NSR, not specifically reported, ALS, advanced life support

^a^, Pooled data from references 6 and 7; additional references supporting the data of the current Table are provided in Additional file 1

^b^, PEA became more frequent than asystole from 2004 and onward

^c^, First column data originate solely from the earliest of the US Registry study (1999–2005)

^d^, As further specified in reference 8 and in the earliest US Registry study (1999–2005)

^e^, Data originate from 172 control patients (missing, n = 18) and 165 VSE group patients (missing, n = 13)

^f^, Time to rhythm analysis was not directly collected in the VSE studies; however, according to the standard application of the ALS algorithm, cardiac arrest rhythm was to be assessed within 1 min of arrival of the resuscitation team; consequently, for the purpose of the current, comparative presentation, time to rhythm analysis was estimated for the VSE studies by adding 1 min to the (directly collected) time to resuscitation team arrival (or time to ALS initiation); this median estimate of 3 min (from collapse to rhythm analysis) seems to be more likely to be associated with asystole rather than PEA, according to the (above-presented) results of the Danish Registry study

^g^, In the earliest US Registry study (see also Additional file 1), 7% of included patients with nonshockable rhythms did not receive epinephrine during resuscitation; notably, in the same study, any use of epinephrine was associated with approximately threefold lower probability of survival to hospital discharge

^h^, The high incidence of PEA in the US and Danish studies is consistent with the speculation that a substantial proportion (e.g. 20–30%) of the patients actually had pseudo PEA, which is associated with increased rates of return of spontaneous circulation (ROSC) and survival to hospital discharge; a systematic review (citation provided in Additional file 1) reported a pseudo-PEA incidence of 56% among 777 PEA patients from 11 observational studies

^i^, Nonmonitored cardiac arrest, longer time to rhythm analysis, arrest within 23.00–07.00 h and unwitnessed arrest were associated with a higher probability of asystole in the Danish Registry study

^k^, Older age (e.g. > 90 years), female sex, overweight/obesity, COPD and gastrointestinal cancer were associated with higher probability of asystole in the Danish Registry study; in the same study, ischemic heart disease and intubation before cardiac arrest were associated with higher probability of PEA

^l^, Reported body mass index was similar in the Greek VSE RCTs and the Danish VAM RCT

^**m**^, Data were reported as duration of resuscitation in the US Registry study and as duration of ALS (i.e. time from resuscitation team arrival to ROSC or ALS termination) in the Greek VSE studies

^n^, Pertinent, supporting references are listed in Additional file 1

^o^, Failed resuscitation indicates irreversibility of the underlying acute pathophysiology, which is consistent with more severe disease; disease severity and poor prognosis frequently contribute to do-not-resuscitate decisions in the US and Denmark, but not in Greece (primarily because of the absence of specific end-of-life legislation)

^p^, As reported above, in the control group of the Greek VSE RCTs, failed resuscitation was associated with very high rates (of 80–90%) of asystole, especially among ICU patients; in such severely ill patients, ALS is substantially more likely to be withheld in the US and Denmark as compared to Greece

Regarding VSE practicability, vasopressin/methylprednisolone physical/chemical stability in normal saline solutions has been previously confirmed [6]. Routinely using prefilled syringes for prompt VSE-administration is feasible/effective [6, 7].

## Conclusion

In view of the above-presented discussion/evidence and until publication of new evidence from a large, ongoing Swedish RCT (www.clinicaltrials.gov/study/NCT05139849), we suggest that VSE [6, 7] might be considered in IHCA [15]. However, the frailty/fragility of the corrected meta-analysis results needs to be considered.

### Electronic supplementary material

Below is the link to the electronic supplementary material.

Supplementary file1 (DOCX 25 kb)

## Declarations

### Availability of data and materials

All data generated or analysed can be made available upon request after publication through a collaborative process. Researchers should provide a methodologically sound proposal with specific objectives in an approval proposal. Please contact the corresponding author for additional information.
