# Correction: A critical reappraisal of vasopressin and steroids in in-hospital cardiac arrest

**DOI:** 10.1186/s13054-024-05043-6

**Published:** 2024-08-15

**Authors:** Spyros D. Mentzelopoulos, Athanasios Chalkias

**Affiliations:** 1 First Department of Intensive Care Medicine, Medical School, National and Kapodistrian University of Athens, Athens, Greece; 2 Department of Intensive Care Medicine, Evaggelismos General Hospital, 45‑47 Ipsilandou St, 10675 Athens, Greece; 3 Institute for Translational Medicine and Therapeutics, University of Pennsylvania Perelman School of Medicine, Philadelphia, PA 19104‑5158 USA; 4 Outcomes Research Consortium, Cleveland, OH 44195 USA

**Correction: Mentzelopoulos and Chalkias Critical Care (2024) 28:191** 10.1186/s13054-024-04962-8

Following publication of the original article [1], the authors identified an error within row 7 of Table 2. In Table 2, row 7, the lowest percentages of postresuscitation hypotension (i.e. 17% and 15%) actually correspond to the intervention group(s) and the highest (i.e. 28% and 29%) to control.

Table 2 row 7 currently reads:

**Table:** 

Lowest MAP ≤ 50 mmHg and SAP ≤ 80 mmHg, intervention group(s) versus control (%)	28% versus 17%—*P* = 0.12 and 29% versus 15%; *P* = 0.03^d^	NR but significant difference unlikely^c^

**Table 2:** Key differences between the Greek VSE trials and the Danish VAM IHCA trial

Key characteristic	VSE 1 and 2—pooled data	VAM-IHCA (Danish trial)	Potential effect
Time to study drugs, median (IQR)—min	4 (3–5)	9 (6–12)	Earlier, simultaneous activation of V1A-vasopressin and α1-adrenergic receptors, with maximization of pressor effect and associated probability of prompt ROSC,^a^ likely occurring only in intervention groups (vs. control) of the VSE 1 and 2 RCTs
Epinephrine and Vasopressin started and always given simultaneously	Yes	No	
Median time lag between first dose of epinephrine and study drugs (min)	0	3–4	
Significantly shorter median (IQR) time to ROSC (min) in intervention group(s) versus control	Yes; 14 (7–24) versus 20 (10–30)—*P* < 0.001	No; 16 (12–25) versus 18 (11–31)—*P*-value NR	Attenuated ischemia / reperfusion injury and lower risk of epinephrine-related adverse effects likely present only in intervention groups (vs. control) of the VSE 1 and 2 RCTs
Significantly lower median (IQR) total dose (mg) of epinephrine in intervention group(s) versus control	Yes; 4 (2–6) versus 5 (3–9)—*P* < 0.001	No; 3 (2–5) versus 3 (2–5)—*P*-value NR	
SAP < 90 mmHg within 20 min of ROSC, intervention group(s) versus control (%)	19% versus 44%—*P* < 0.001^b^	NR but significant difference unlikely ^c^	Early postresuscitation hypotension has been consistently associated with increased in-hospital mortality [11]; reported differences may partly explain the long-term benefit observed only in the VSE 1 and 2 RCTs
Lowest MAP ≤ 50 mmHg and SAP ≤ 80 mmHg, intervention group(s) versus control (%)	28% versus 17%—*P* = 0.12 and 29% versus 15%; *P* = 0.03^d^	NR but significant difference unlikely^c^	
Postresuscitation use of steroids in intervention group(s) versus control (%)	99% versus 22% ^d^	26% versus 46% ^e^	In VAM-IHCA, there was non-protocolized, more frequent use of potentially beneficial interventions in the control group
Postresuscitation use of ECMO in intervention group(s) versus control (%)	0% versus 0% ^d,f^	14% versus 30% ^e^	

VSE, vasopressin-steroids-epinephrine; VAM, vasopressin-adrenaline-methylprednisolone; IHCA, in-hospital cardiac arrest; IQR, interquartile range; ROSC, return of spontaneous circulation; NR, not reported; RCT, randomized clinical trial; SAP, systolic arterial pressure; MAP, mean arterial pressure; ECMO, extracorporeal membrane oxygenation

Reported data originate from the total of the VSE 1 and 2 or VAM-IHCA participants, unless otherwise specified. For data reported as median (IQR), *P*-values were determined using the Mann Whitney U test. For data reported as percentages, *P*-values were determined by Fisher’s exact test

^a^, Defined as ROSC after ≤ 3 vasopressor doses

^b^, VSE 1 and 2 data originate from the pooled subgroup of survivors for ≥ 4 h with postresuscitation shock and available SAP data (intervention, n = 94; control, n = 72)

^c^, Analyses of VAM-IHCA hemodynamic data revealed very similar, early postresuscitation arterial pressures and vasopressor support [2]; consequently, there was likely no significant, between-group difference in the frequency of postresuscitation hypotension

^d^, VSE 1 and 2 data originate from the pooled subgroup of survivors for ≥ 24 h (intervention, n = 94; control, n = 69)

^e^, VAM-IHCA data originate from survivors for ≥ 24 h (intervention, n = 63; control, n = 61)

^f^, There was no postresuscitation use of ECMO in the VSE 1 and 2 studies

]

Table 2 row 7 should read:

**Table:** 

Lowest MAP ≤ 50 mmHg and SAP ≤ 80 mmHg, intervention group(s) versus control (%)	17% versus 28%—*P* = 0.12 and 15% versus 29%; *P* = 0.03^d^	NR but significant difference unlikely^c^

**Table 2:** Key differences between the Greek VSE trials and the Danish VAM IHCA trial

Key characteristic	VSE 1 and 2—pooled data	VAM-IHCA (Danish trial)	Potential effect
Time to study drugs, median (IQR)—min	4 (3–5)	9 (6–12)	Earlier, simultaneous activation of V1A-vasopressin and α1-adrenergic receptors, with maximization of pressor effect and associated probability of prompt ROSC,^a^ likely occurring only in intervention groups (vs. control) of the VSE 1 and 2 RCTs
Epinephrine and Vasopressin started and always given simultaneously	Yes	No	
Median time lag between first dose of epinephrine and study drugs (min)	0	3–4	
Significantly shorter median (IQR) time to ROSC (min) in intervention group(s) versus control	Yes; 14 (7–24) versus 20 (10–30)—*P* < 0.001	No; 16 (12–25) versus 18 (11–31)—*P*-value NR	Attenuated ischemia / reperfusion injury and lower risk of epinephrine-related adverse effects likely present only in intervention groups (vs. control) of the VSE 1 and 2 RCTs
Significantly lower median (IQR) total dose (mg) of epinephrine in intervention group(s) versus control	Yes; 4 (2–6) versus 5 (3–9)—*P* < 0.001	No; 3 (2–5) versus 3 (2–5)—*P*-value NR	
SAP < 90 mmHg within 20 min of ROSC, intervention group(s) versus control (%)	19% versus 44%—*P* < 0.001^b^	NR but significant difference unlikely ^c^	Early postresuscitation hypotension has been consistently associated with increased in-hospital mortality [11]; reported differences may partly explain the long-term benefit observed only in the VSE 1 and 2 RCTs
Lowest MAP ≤ 50 mmHg and SAP ≤ 80 mmHg, intervention group(s) versus control (%)	17% versus 28%—*P* = 0.12 and 15% versus 29%; *P* = 0.03^d^	NR but significant difference unlikely^c^	
Postresuscitation use of steroids in intervention group(s) versus control (%)	99% versus 22% ^d^	26% versus 46% ^e^	In VAM-IHCA, there was non-protocolized, more frequent use of potentially beneficial interventions in the control group
Postresuscitation use of ECMO in intervention group(s) versus control (%)	0% versus 0% ^d,f^	14% versus 30% ^e^	

VSE, vasopressin-steroids-epinephrine; VAM, vasopressin-adrenaline-methylprednisolone; IHCA, in-hospital cardiac arrest; IQR, interquartile range; ROSC, return of spontaneous circulation; NR, not reported; RCT, randomized clinical trial; SAP, systolic arterial pressure; MAP, mean arterial pressure; ECMO, extracorporeal membrane oxygenation

Reported data originate from the total of the VSE 1 and 2 or VAM-IHCA participants, unless otherwise specified. For data reported as median (IQR), *P*-values were determined using the Mann Whitney U test. For data reported as percentages, *P*-values were determined by Fisher’s exact test

^a^, Defined as ROSC after ≤ 3 vasopressor doses

^b^, VSE 1 and 2 data originate from the pooled subgroup of survivors for ≥ 4 h with postresuscitation shock and available SAP data (intervention, n = 94; control, n = 72)

^c^, Analyses of VAM-IHCA hemodynamic data revealed very similar, early postresuscitation arterial pressures and vasopressor support [2]; consequently, there was likely no significant, between-group difference in the frequency of postresuscitation hypotension

^d^, VSE 1 and 2 data originate from the pooled subgroup of survivors for ≥ 24 h (intervention, n = 94; control, n = 69)

^e^, VAM-IHCA data originate from survivors for ≥ 24 h (intervention, n = 63; control, n = 61)

^f^, There was no postresuscitation use of ECMO in the VSE 1 and 2 studies

]

Table 2 has been updated in this correction and the original article [1] has been corrected.
