# Supplementary material for: A critical reappraisal of vasopressin and steroids in in-hospital cardiac arrest
Source: Crit Care. 2024 Jun 6;28:191. doi: 10.1186/s13054-024-04962-8 (PMC11155013; doi:10.1186/s13054-024-04962-8)
Supplement: Supplementary file 1 — Supplementary file1 (DOCX 25 kb) [file 13054_2024_4962_MOESM1_ESM.docx]

**Supplementary Online Content**

**A Critical Reappraisal of Vasopressin and Steroids in In-hospital Cardiac Arrest**

**Authors**

Spyros D. Mentzelopoulos, MD, PhD^1^, Athanasios Chalkias, MD, PhD^2,3^

**Department(s) and institution(s)**

^1^ First Department of Intensive Care Medicine, Medical School, National and Kapodistrian University of Athens, Athens, Greece

^2^ Institute for Translational Medicine and Therapeutics, University of Pennsylvania Perelman School of Medicine, Philadelphia, PA 19104-5158, USA

^3^ Outcomes Research Consortium, Cleveland, OH, 44195, USA

This supplementary material has been provided by the authors to give readers additional information about their work.

# Additional references supporting the data presented in Table 3

# A] Data column 1, United States Registry studies 1999-2000

1. Meaney PA, Nadkarni VM, Kern KB, Indik JH, Halperin HR, Berg RA. Rhythms and outcomes of adult in-hospital cardiac arrest. Crit Care Med. 2010;38:101-8.
2. Girotra S, Nallamothu BK, Spertus JA, Li Y, Krumholz HM, Chan PS; American Heart Association Get with the Guidelines–Resuscitation Investigators. Trends in survival after in-hospital cardiac arrest. N Engl J Med. 2012;367:1912-20.
3. Thompson LE, Chan PS, Tang F, et al; American Heart Association’s Get With the Guidelines-Resuscitation Investigators. Long-Term Survival Trends of Medicare Patients After In-Hospital Cardiac Arrest: Insights from Get With The Guidelines-Resuscitation^®^. Resuscitation. 2018;123:58-64.

**B] Data columns 1 and 3, United States Registry study 2006-2019 and Danish Registry study 2017-2018**

1. Andrea L, Shiloh AL, Colvin M, Rahmanian M, Bangar M, Grossestreuer AV, Berg KM, Gong MN, Moskowitz A; American Heart Association's Get With The Guidelines®-Resuscitation Investigators. Pulseless electrical activity and asystole during in-hospital cardiac arrest: Disentangling the 'nonshockable' rhythms. Resuscitation. 2023;189:109857.
2. Høybye M, Stankovic N, Lauridsen KG, Holmberg MJ, Andersen LW, Granfeldt A. Pulseless electrical activity vs. asystole in adult in-hospital cardiac arrest: Predictors and outcomes. Resuscitation. 2021;165:50-57.

**C] Systematic review mentioned in footnote "h"**

1. Wu C, Zheng Z, Jiang L, et al. The predictive value of bedside ultrasound to restore spontaneous circulation in patients with pulseless electrical activity: A systematic review and meta-analysis. PLoS One. 2018;13:e0191636.

**D] Articles reporting on legal support and documentation of resuscitation decisions, and on frequency of failed resuscitation in the United States, Greece and Denmark.**

1. Prendergast TJ, Luce JM. Increasing incidence of withholding and withdrawal of life support from the critically ill. Am J Respir Crit Care Med. 1997;155:15-20.
2. Shore S, O'Leary M, Kamdar N, Harrod M, Silveira MJ, Hummel SL, Nallamothu BK. Do Not Attempt Resuscitation Order Rates in Hospitalized Patients With Heart Failure, Acute Myocardial Infarction, Chronic Obstructive Pulmonary Disease, and Pneumonia. J Am Heart Assoc. 2022;11:e025730.
3. Kranidiotis G, Gerovasili V, Tasoulis A, Tripodaki E, Vasileiadis I, Magira E, Markaki V, Routsi C, Prekates A, Kyprianou T, Clouva-Molyvdas PM, Georgiadis G, Floros I, Karabinis A, Nanas S. End-of-life decisions in Greek intensive care units: a multicenter cohort study. Crit Care. 2010;14:R228.
4. Mentzelopoulos SD, Bossaert L, Raffay V, Askitopoulou H, Perkins GD, Greif R, Haywood K, Van de Voorde P, Xanthos T. A survey of key opinion leaders on ethical resuscitation practices in 31 European Countries. Resuscitation. 2016;100:11-7.
5. Christensen L, Jensen H, Kristensen S, Goldinger M, Gjedsted J, Christensen S, Sprung C, Avidan A, Mentzelopoulos SD, Bulow HH. Treatment limitations in intensive care units. Dan Med J. 2021;68:A03210235.
6. Avidan A, Sprung CL, Schefold JC, Ricou B, Hartog CS, Nates JL, Jaschinski U, Lobo SM, Joynt GM, Lesieur O, Weiss M, Antonelli M, Bülow HH, Bocci MG, Robertsen A, Anstey MH, Estébanez-Montiel B, Lautrette A, Gruber A, Estella A, Mullick S, Sreedharan R, Michalsen A, Feldman C, Tisljar K, Posch M, Ovu S, Tamowicz B, Demoule A, DeKeyser Ganz F, Pargger H, Noto A, Metnitz P, Zubek L, de la Guardia V, Danbury CM, Szűcs O, Protti A, Filipe M, Simpson SQ, Green C, Giannini AM, Soliman IW, Piras C, Caser EB, Hache-Marliere M, Mentzelopoulos SD; ETHICUS-2 Study Group. Variations in end-of-life practices in intensive care units worldwide (Ethicus-2): a prospective observational study. Lancet Respir Med. 2021;10:1101-1110.
7. Mentzelopoulos SD, Chen S, Nates JL, Kruser JM, Hartog C, Michalsen A, Efstathiou N, Joynt GM, Lobo S, Avidan A, Sprung CL; End-of-life Practice Score Study Group. Derivation and performance of an end-of-life practice score aimed at interpreting worldwide treatment-limiting decisions in the critically ill. Crit Care. 2022;26:106.
